# Pelvic Abscess Caused by Ureteral Calculus and Abscess Treatment through Aspiration by Transperineal Puncture

**DOI:** 10.1155/2024/1723185

**Published:** 2024-01-25

**Authors:** Bo-Ran An, Chao Gao, Di An

**Affiliations:** ^1^Gastroenterology Department, Affiliated Hospital of Hebei University, No. 212 Yuhua East Road, Baoding, Hebei Province 071000, China; ^2^2nd Ward, Department of Urology, Affiliated Hospital of Hebei University, No. 212 Yuhua East Road, Baoding, Hebei Province 071000, China; ^3^Department of Neurology, Affiliated Hospital of Hebei University, No. 212 Yuhua East Road, Baoding, Hebei 071000, China

## Abstract

Pelvic abscess is mostly caused by gynecological inflammation or digestive system diseases such as appendicitis or Crohn's disease. This case of pelvic abscess originates from ureteral calculus and is not commonly seen in clinical practice. This is mainly due to the patient's ureteral stones not being actively treated. After local puncture and pus extraction, as well as the application of effective antibiotics, the patient recovered. Therefore, this case provides clinical doctors with experience that ureteral stones may cause serious complications and should be actively treated after detection.

## 1. Introduction

Pelvic abscess is mostly caused by female pelvic inflammation while digestive system diseases such as appendicitis and Crohn's disease can also cause it [1, 2]. It is rare that pelvic abscess is caused by ureteral calculus, while it can cause abdominal abscess in some cases. There is no literature report on pelvic abscess caused by ureteral calculus so far. Complete urinary tract obstruction caused by ureteral calculus can cause renal atrophy if not treated in time because of high pressure in the renal pelvis [3]. Sometimes, aerobic or anaerobic bacteria are detected as single flora in an abscess while mixed bacterial infection also exists. It can be guided that of puncture of pelvic abscess by imaging system such as B-ultrasound or CT. The puncture approach can be selected through abdominal, rectal, or buttocks, and women can also be selected through vaginal vault. Postoperative treatment with antibiotics often has good therapeutic effects. This patient underwent transperineal puncture and received postoperative antibiotic treatment according to bacterial culture and drug sensitivity test and have recovered.

## 2. Case Presentation

**Figure 1:**
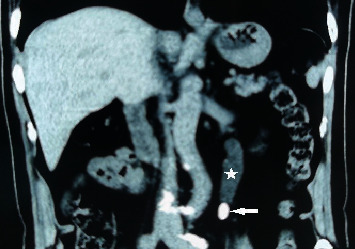
Left ureteral effusion (star), with calculus completely obstructing the urinary tract (white arrow).

**Figure 2:**
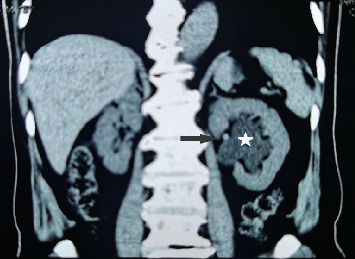
Left renal pelvis effusion (star) with visible small stone shadow (gray arrow).

**Figure 3:**
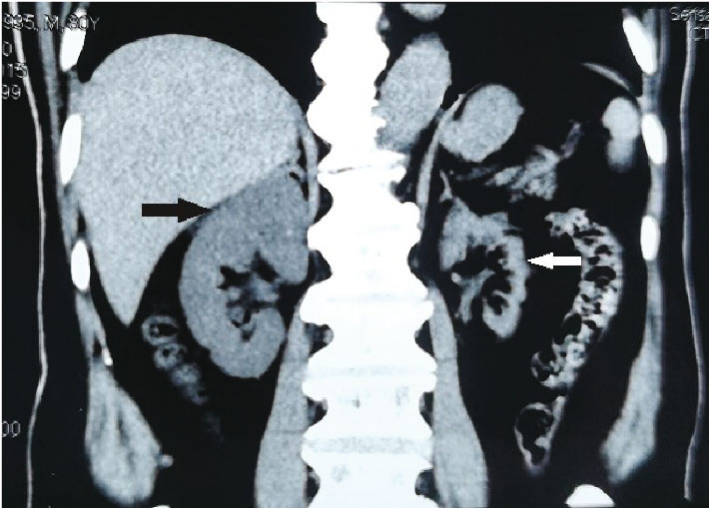
CT shows left renal atrophy, renal cortex thinning (white arrow), and right kidney compensatory hypertrophy (black arrow).

**Figure 4:**
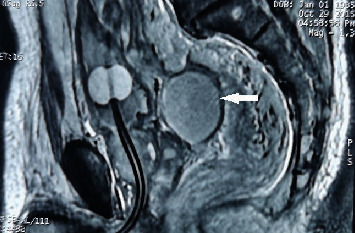
A mixed signal shadow (white arrow) can be seen as a quasielliptical on MRI T2WI, located between the rectum and bladder. The left high signal shadow (hollow in middle) is the water bag of the Foley catheter located in the bladder at the inner mouth of the urethra.

**Figure 5:**
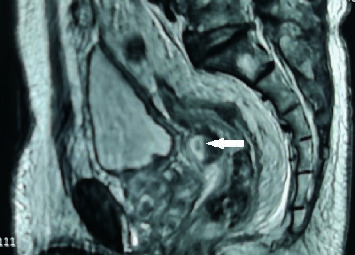
Abscess shrank with low signal in its center (white arrow) after MRI reexamination.

The patient, male, 88 years old, was admitted to our hospital on February 2013 due to abdominal pain and fever. He said he had history of hypertension and atrial fibrillation for many years, but neither appendicitis nor Crohn's disease in his life span when we inquired his medical history. Laboratory investigations revealed an elevated white blood cell count. CT examination revealed that left ureteral calculus was located in the middle segment of the ureter at the umbilical level, left ureter effusion in its middle and upper part, and left renal pelvis hydronephrosis continuously to ureter effusion (Figures 1 and 2) after admission. His pain disappeared on his abdomen after medication care. He was administrated with antibiotics due to elevated white blood cells, elevated CRP, and faster erythrocyte sedimentation rate during hospitalization in 2013, and inflammatory indicators recovered upon discharge. He refused to undergo ureteral calculus surgery or lithotripsy treatment. The patient often complained he had recurrent abdominal pain and low fever (37-38°C) after discharge. He used antibiotics intermittently for 2 years that he often bought himself from a drug store after consulting local general practitioner, which sometimes could alleviate his symptoms. On October 2015, he was admitted again due to abdominal pain and fever. His temperature was 37.8°C high, but pulse rate and blood pressure were normal. Laboratory results showed leucocytosis (12.3 × 10^9^/L, neutrophil 78%, and 22% lymphocytes), increased erythrocyte sedimentation rate (33 mm/h), and C-reactive protein concentration (45 mg/L), but his kidney function was normal. The left kidney had atrophied, and the renal cortex became thinner (Figure 3) when examined by B-ultrasound and CT after his admission. Fluid density shadow was found in the pelvic cavity by B-ultrasound examination. MRI examination suggested mixed signal shadows in the pelvic cavity on T2WI, considering pelvic abscess (Figure 4). Transperineal approach for abscess puncture was performed that guided by transrectal B-ultrasound since his diagnosis had been confirmed by MRI 4 days before. The abscess puncture point was chosen in accordance with the needle insertion point of prostate biopsy because the abscess was posterior to the prostate. The puncture place was the midpoint between the center of the perineum and the anus. The patient took the bladder lithotomy position, the skin was disinfected regularly, and local infiltration anesthesia was undergone. A small incision was made at the puncture point before the operation with a surgical knife. The puncture needle was held in the right hand to puncture at the incision point and operated to the center of the abscess under the guidance of ultrasound [4]. The puncture needle was severed by BARD Medical Technology (Shanghai) Co., Ltd. The type was Bard Max Core, Disposable Core biopsy instrument, 18 gauge, 22 cm long. The B-ultrasound instrument was a product by BK Medical Group Company, and the type was flex focus ultrasound scanner 1202. The needle core was withdrawn after successful puncturing, and connected a syringe to extract the pus. About 40 ml of pus was extracted during aspiration. Ornidazole and levofloxacin injections were used to flush the purulent cavity after operation, and liquid medicine mixture was injected for retention after flushing. The needle core was inserted into the puncture needle, and it was removed after pus extraction. Subsequently, the same medications were administered intravenously. Most of the abscess disappeared in follow-up MRI (Figure 5) after 3 days of treatment. The bacterial culture results showed that *Escherichia coli* grew in aerobic culture after pus extraction. Susceptibility testing revealed that many antibacterial drugs were effective to this strain of *Escherichia coli*, including cefoperazone/sulbactam, ticarcillin/clavulanate potassium, piperacillin/tazobactam, cefoxitin, cefuroxime, ceftriaxone, cefotaxime, cefepime, imipenem, meropenem, amikacin, levofloxacin, aztreonam, and chloramphenicol. Anaerobic culture had bacterial growth, but the species of bacteria had not been identified, and no antianaerobic drug sensitivity experiments were conducted yet. Leucocytes, erythrocyte sedimentation rate (33 mm/h), and C-reactive protein were re-examined before his discharge. All of them decreased but had not reached the normal level. Antibiotics were selected based on the results of antimicrobial drug sensitive test combined with antianaerobic medicine, which were levofloxacin and ornidazole. Antibiotics are used lasting for 3 weeks, including hospitalization and after discharge period. The patient follow-up had generally been in good condition in half a year, and the pelvic abscess had not recurred. He recently came to our hospital again and said that he had not experienced pelvic pain or low fever in the past 8 years again since he was discharged in 2015. Therefore, we believe that his pelvic abscess has been completely cured.

## 3. Discussion

There are many causes for pelvic abscess, most of which is mostly caused by delayed treatment of gynecological inflammatory or appendicitis perforation, Crohn's disease, etc. Gynecological inflammation can cause fallopian tube abscess [5], while Miller tube infection can cause pelvic abscess during pregnancy in women [6]. Pelvic abscess caused by urinary system diseases can also be urinary tract diverticulum infection in addition to ureteral stones like this case [7]. Exogenous foreign bodies have also been reported in literature [8]. Some uncommon factors may also lead to the formation of pelvic abscess. The causes of the disease can be traced back, such as fish bones [8], while the infection pathway of some pelvic abscess cannot be traced back [9]. There was infection caused by Streptococcus pneumoniae that occurred in the retroperitoneum, causing infection in the iliopsoas and gluteus muscles. But pelvic abscesses have common characteristics, namely, pain and fever, regardless of their various reasons. The symptoms may vary according to the abscess that occurred in different parts of the pelvic. For example, abscess present in the pelvic muscles can cause abnormalities in the movement system [10].

There are currently no reports of pelvic abscess caused by ureteral calculus. The ureteral stone in this patient was located at the level of the umbilicus in the middle segment of the ureter. Ureteral stones are easily embedded in the three narrow parts of the ureter, and the ureteral stone was embedded above the second narrow part of the ureter in this patient. The formation of the patient's pelvic abscess may be related to the factors that the infection in left ureter and renal pelvis was caused by ureteral calculus resulted in necrosis of the ureteral wall, causing left ureter rupture and forming ureteroperitoneal fistula, leading to the formation of an abscess in the lower part of left abdomen, because no left ureteral calculus and no left hydronephrosis were found after the patient's hospitalization in 2015. If it was not treated in time that complete upper urinary tract obstruction had been caused by ureteral calculus, pyonephrosis might occur due to infection. Although the kidney is a retroperitoneal organ, pyonephrosis can also cause abdominal abscess [11]. Due to the influence of gravity, the pus which was discharged into the abdominal cavity (which may form a pseudocapsule) might gradually migrate to the pelvic area, causing a pelvic abscess.

The left renal cortex of the patient became thinner which was shown at the abdominal CT of the patient in 2015, indicating that there is still some residual function of urine excretion in the left kidney. It is considered that increased pressure in the renal pelvis and long-term pressure on the renal cortex can lead to kidney atrophy. It is speculated that the patient's ureteral rupture healed after his pus and stone ureter discharged into cavities. If the left ureter rupture does not heal, urine filtered by the left kidney could go into the abdominal cavity, which often leads to peritonitis [12]. This patient did not frequently experience peritonitis after he was discharged in 2015. Peritonitis caused by urinary extravasation has also been reported in literature.

There is no recurrence of patient's pelvic abscess after he was discharged since 2015. There are no treatment measures having been taken for ureteral stones. We are considering ureteral stones and abscesses migrating to the pelvic cavity together. After the abscess is cured, we think pelvic stone still remains in the pelvic cavity.

Surgery or aspiration treatment is often required after the formation of pelvic abscess. At present, the methods of aspiration treatment include such as abdominal wall approach, anterior rectal wall approach, and gluteus muscle approach. Women can also use vaginal vault approach. It often required B-ultrasound or CT guidance during puncture to improve the accuracy. There has been report of using digestive endoscopy technology to treat deep pelvic abscess in recent years [13]. As to this patient's pelvic abscess, we first considered transrectal puncture treatment under B-ultrasound guidance. The transrectal puncture approach was limited due to the high location of the patient's abscess. And the puncture operation was successfully completed when we used transrectal ultrasound guidance and adopted a transperineal approach [14].

The bacteria are often *Escherichia coli*, which cause pyogenesis in the renal pelvis and ureter induced by ureteral stones. Although current literature suggests that *Escherichia coli* have a high resistance rate to levofloxacin, some strains are still sensitive to levofloxacin. This is the reason why we injected levofloxacin into the pus cavity after extracting treatment, which is also based on experience in medication. After the patient was discharged from our hospital where the symptoms of ureteral stone had alleviated in 2013, the patient had used antibiotics intermittently for long term due to frequent abdominal pain and low fever, which may lead possibly for dysbacteriosis to occur. Pelvic abscess caused by anaerobic bacteria has also been reported [15]. This was why we injected ornidazole into the pus cavity targeting at anaerobic bacteria. Due to the fact that the patient's inflammatory indicators did not recover upon discharge, the patient was administered to continue using antibiotics after his discharge. Levofloxacin was administered further to the patient based on the bacterial culture results after his discharge. However, anaerobic cultivation had bacterial growth, but laboratory condition was not enough to fulfill the drug sensitivity test for anaerobic bacteria. Therefore, we choose to continue using ornidazole therapy for anaerobic bacteria. After the patient finished his course of treatment, the patient did not experience any lower abdominal pain or low fever similarly to abscess treatment ago, for a total follow-up of 6 months. It was considered that there was no recurrence of pelvic abscess within 6 months.

Nephrectomy can also be considered as a treatment option for pyonephrosis caused by ureteral calculi. Due to the patient's underlying diseases such as hypertension and atrial fibrillation, he might not be able to tolerate surgery, and the patient had no intention for surgery. Therefore, the treatment method of nephrectomy was not considered. After the patient's pelvic abscess was cured, the pelvic stone was not treated, and the patient did not experience any recurrence of pelvic abscess, indicating that the ureteral calculus of the patient was noninfectious, because antibacterial drugs can hardly penetrate into the stones.

## 4. Conclusion

From this case, clinical doctors can be reminded that ureteral calculi, especially those that cause complete obstruction of the ureter, can not only cause kidney damage but also cause pelvic abscess. Doctors should alert to the possibility of pelvic abscess formation for patients who frequently experience pelvic pain and fever. A detailed medical history should be investigated for patients with pelvic abscess to identify the cause of it due to the various etiologies. Antibiotic target on both aerobic and anaerobic should be administered after abscess aspiration because mixed flora might exist in an abscess. Transperineal puncture is also another approach for male patients based on the successful experience of this patient and previous literature.

## Data Availability

The data and radiology images used are available from the corresponding author upon reasonable request.
